# The Optimal Cut-Points of Alanine Aminotransferase for Screening Metabolic Syndrome in Iranian Adults

**DOI:** 10.5812/ijem-151542

**Published:** 2025-01-25

**Authors:** Samaneh Asgari, Fereidoun Azizi, Farzad Hadaegh

**Affiliations:** 1 Prevention of Metabolic Disorders Research Center, Research Institute for Endocrine Sciences, Shahid Beheshti University of Medical Sciences, Tehran, Iran; 2 Endocrine Research Center, Research Institute for Endocrine Sciences, Shahid Beheshti University of Medical Sciences, Tehran, Iran

**Keywords:** Alanine Aminotransferase, Cut-Off Point, Prevalence, Metabolic Syndrome

## Abstract

**Background:**

Studies have reported that the activity of alanine aminotransferase (ALT) is a key biomarker for screening liver cell damage, such as non-alcoholic fatty liver disease (NAFLD).

**Objectives:**

Since individuals with metabolic syndrome (MetS) are at high risk for NAFLD, we aimed to determine gender-specific ALT thresholds for screening MetS in the Tehranian population.

**Methods:**

We conducted a cross-sectional study from 2018 to 2022, involving 4,968 adults aged 20 - 70 years (2,732 females). Multivariable logistic regression analysis was performed to assess the association between ALT levels and the prevalence of MetS, as well as its individual components. Additionally, gender-specific ALT cut-off points were determined using the maximum Youden's Index. The area under the receiver operating characteristic curve (AUC) was calculated to derive thresholds and compare them with the previously introduced cut-off points for liver-related mortality in the U.S. population (US-LRM) (ALT > 19 U/L for females, > 29 U/L for males). We also examined the diagnostic performance of the derived cut-off points in 11 147 individuals (7,154 women) from the atherosclerosis risk in communities (ARIC) study as an external validation.

**Results:**

The odds ratio (OR) from the logistic regression analysis showed that each 5 U/L increase in ALT level was associated with an increased prevalence of MetS [19% for females and 8% for males] and its components (ranging from 7 - 19% in females and 3-10% in males; all P-values < 0.05). The suggested cut-off point for ALT in males was 21 U/L, with a sensitivity of 72.1% and specificity of 47.1%. For females, with a threshold of 18 U/L, the corresponding values were 57.9% sensitivity and 66.5% specificity. Compared to the US-LRM suggested cut-off points in the US population, the AUC of our suggested threshold increased in males (60% vs. 56%, respectively), while for females, it remained the same as in the pretest (≈ 62%). Using ARIC data, our suggested threshold showed nearly identical AUC values to the US-LRM threshold in females (58% vs. 57%, respectively), whereas for males, the highest AUC was observed for our suggested cut-off points (56%), followed by the mortality-related threshold (53%).

**Conclusions:**

The cut-off point for screening MetS among Iranian women was almost identical to the lower suggested threshold in American guidelines but was notably lower for defining abnormal ALT levels in males.

## 1. Background

Metabolic syndrome (MetS), which encompasses various metabolic abnormalities, including high blood pressure, obesity, dyslipidemia, and high blood glucose, is a global health challenge affecting 14 - 32% of the world’s population (1), with an overall prevalence of 30.4% among Iranian adults (2). Individuals with MetS are at high risk for type 2 diabetes (T2DM), chronic kidney disease (CKD), non-alcoholic fatty liver disease (NAFLD), and cardiovascular events (3-6). 

Several studies have reported that the activity of alanine aminotransferase (ALT) is a key biomarker for screening liver cell damage, such as NAFLD (7-12). Hepatocellular damage associated with MetS can elevate ALT levels, leading to a positive correlation (13-15). A meta-analysis indicated that for each 5 U/L increase in serum ALT, the risk of adult treatment panel III (ATP III) defined MetS increased by 10%. Furthermore, ALT levels exceeding 33.4 U/L were associated with a more than 60% higher risk of MetS, with low heterogeneity observed across the included studies (16). 

A cross-sectional study in the Korean population reported that ALT elevation from 16 U/L to 27 U/L in men and from 11 U/L to 19 U/L in women more than doubled the risk of MetS (17). In another study conducted among Chinese individuals aged > 35 years, an increase in ALT levels from 11.4 U/L to 29.5 U/L was associated with a 1.9-fold increase in the risk of MetS. However, an ALT ≥ 20.1 U/L was suggested as the recommended threshold for the diagnosis of MetS (sensitivity 76.8%, specificity 81.4%) (18). A recent cross-sectional study in the southeastern region of Iran found that elevated ALT (> 40 U/L in males and > 35 U/L in females) increased the odds of ATP III-defined MetS by 46% (19). In another study conducted in northern Iran, elevated ALT (> 40 U/L for both males and females) increased the odds of IDF-defined MetS by 74% in males, while this association was not significant among females (20). 

Despite clear evidence of a positive association between serum ALT levels and MetS, selecting the optimal threshold for elevated ALT remains challenging due to the potential impact of ethnicity, gender, and varying definitions of MetS. Iran, located in the Middle East and North Africa region, has a high incidence and prevalence of MetS. However, data on appropriate elevated ALT cut-off points for the Iranian population are limited (19, 20). 

## 2. Objectives

Therefore, the aim of the current study was twofold: First, to determine gender-specific ALT thresholds for screening MetS among Tehranian adults; and second, to examine the diagnostic performance of the derived cut-off points in the American population.

## 3. Methods 

### 3.1. Study Population 

The Tehran lipid and glucose study (TLGS) is a longitudinal study conducted on an urban population in Tehran, with participants aged ≥ 3 years. The study aims to determine the prevalence and incidence of non-communicable diseases (NCDs) and related risk factors. It is a community-based study that recruited volunteers in two phases: The first phase, from 1999 to 2001, involved 15,005 participants, while the second phase, from 2001 to 2005, involved 3,550 participants. The study follows a tri-annual interval design and is planned to continue for at least 20 years, with five additional phases conducted from 2005 to 2022 (phase 3: 2005 - 2008; phase 4: 2008 - 2011; phase 5: 2012 - 2015; phase 6: 2015 - 2018; and phase 7: 2018 - 2022). The design and methodology of the TLGS have been previously reported (21). 

**Figure 1.**
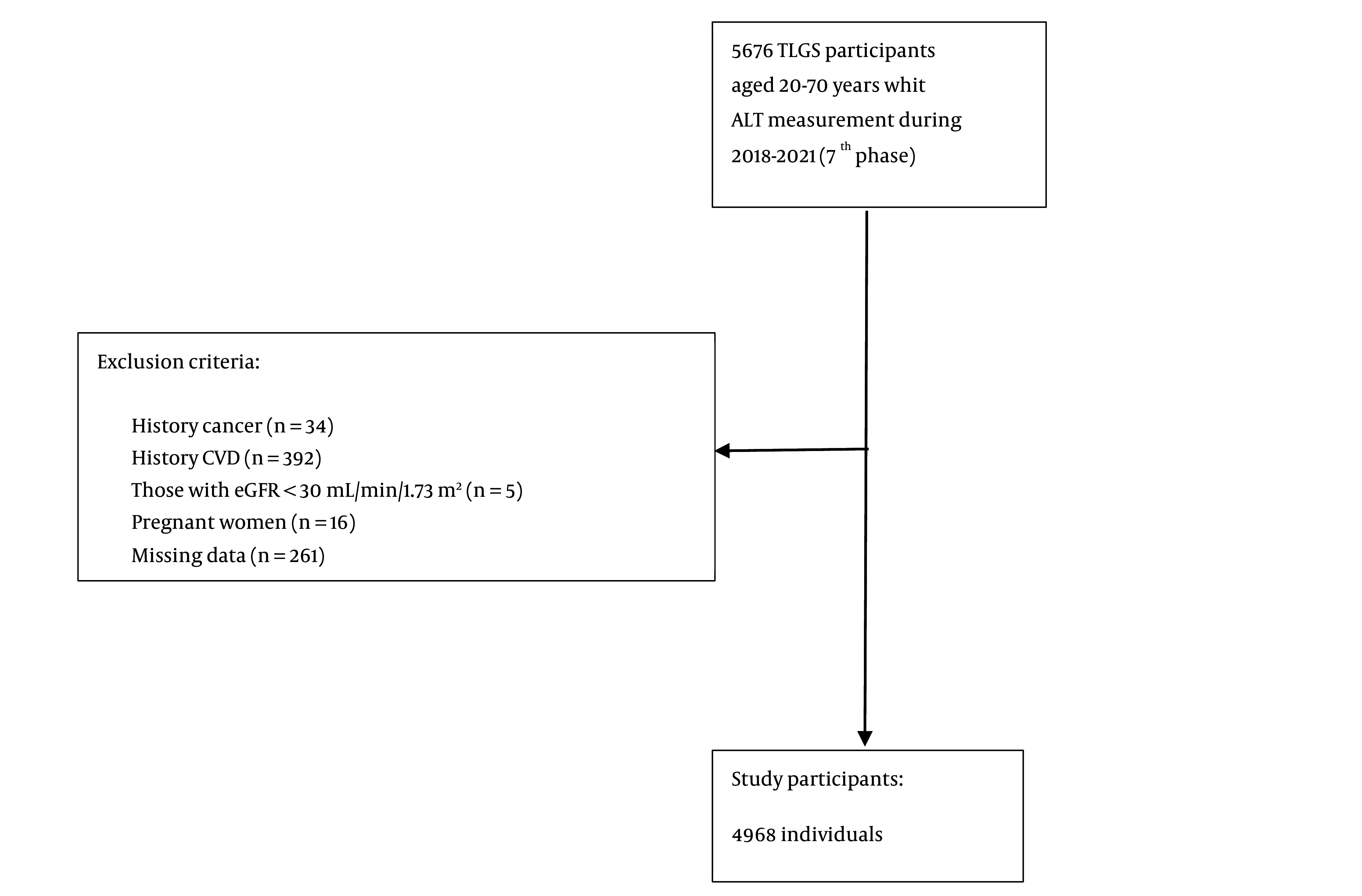
Flow chart of study participants: Tehran lipids and glucose study (TLGS) 2018 - 2021.

For the current study, adults aged 20 - 70 years who had ALT measurements taken between 2018 and 2021 (n = 5,676) were included. Individuals with a history of cancer (n = 34), cardiovascular diseases (n = 392), those undergoing hemodialysis (n = 5) (eGFR < 30 mL/min/1.73 m²), and pregnant women (n = 16) were excluded from the study. After removing 261 individuals with insufficient data on MetS and variables such as marital status, education level, smoking habits, and physical activity, 4,968 individuals (2,732 women) were included in the study (Figure 1). 

We also aimed to validate the suggested cut-points in the American population. To this end, we considered the atherosclerosis risk in communities (ARIC) study (22), which recruited participants from the American population. Of the total 12,947 individuals aged 45 - 64 years who participated in the second phase of the study (1990 - 1992) and had available information on ALT, after applying the exclusion criteria (n = 1 800), 11,147 individuals (7,154 women) were included in the current study. This study approved by the Institutional Review Board (IRB) of the Research Institute for Endocrine Sciences (RIES), Shahid Beheshti University of Medical Sciences, Tehran, Iran (Ethic code: IR.SBMU.ENDOCRINE.REC.1402.022)., and all participants provided informed consent.

### 3.2. Clinical and Laboratory Measurements 

Trained interviewers conducted assessments on various aspects, including demographic information, family history of diabetes (FH-DM), cardiovascular disease (CVD) history, medication history, education level, and smoking habits. The participants' anthropometric measurements were taken while they were wearing light clothing and no shoes. Weight was measured using a digital scale (Seca 707, Seca Corp; range: 0.1 - 150 kg, sensitivity: 0.1 kg). Height was measured while standing with the shoulders in normal alignment, using a tape measure. Waist circumference (WC) at the umbilical level was measured using an unstretched tape measure. 

After a 15-minute rest, systolic and diastolic blood pressures (SBP and DBP) were measured twice on the right arm (with a 5-minute interval) using a standardized mercury sphygmomanometer (calibrated by the Iranian Institute of Standards and Industrial Research). The average of the two readings was considered as the blood pressure measurement. 

A venous blood sample was collected from all subjects after 12 - 14 hours of overnight fasting. Fasting plasma glucose (FPG) was measured using an enzymatic colorimetric method with glucose oxidase. High-density lipoprotein cholesterol (HDL-C) was measured using a homogeneous method (HDL-C Immuno FS). Triglycerides (TG) were assayed using an enzymatic colorimetric method with glycerol phosphate oxidase. The coefficients of variation (CVs) for glucose were 2.2% for both intra- and inter-assay measurements. The intra-assay and inter-assay CVs for HDL cholesterol were 0.5% and 2%, respectively. Triglycerides had intra-assay and inter-assay CVs of 0.6% and 1.6%, respectively. 

The kinetic colorimetric Jaffe method was used to assay serum creatinine (SCr) levels. At the same time, ALT was measured using an optimized UV test according to the International Federation of Clinical Chemistry and Laboratory Medicine (IFCC) on a photometry system. The intra-assay and inter-assay CVs for ALT were 2.2% and 3.8%, respectively. Commercial kits (Pars Azmoon Inc., Tehran, Iran) and a Selectra 2 auto-analyzer (Vital Scientific, Spankeren, The Netherlands) were used to perform the analysis in the TLGS research laboratory on the same day as blood sampling. To ensure measurement quality, assayed serum controls in two different concentrations (TruLab N and TruLab P; Pars Azmoon Inc.) were used.

### 3.3. Variable Definitions 

Body Mass Index was calculated as weight (kg) divided by the square of height (m²). Marital status was classified into three categories: Single (used as the reference group), married, or widowed/divorced. Education level was divided into three categories: (1) < 6 years (used as the reference group), (2) 6 - 12 years, and (3) ≥ 12 years. Smoking habits were categorized into three groups: (1) Current smokers, which included participants who smoked cigarettes daily or occasionally, as well as those who used water pipes or hookahs; (2) past smokers, which included participants who had smoked in the past; and (3) non-smokers (used as the reference group). Physical activity data were collected using the Modifiable Activity Questionnaire (MAQ) (23, 24). The average metabolic equivalent of task (MET) score was used to measure physical activity and was categorized into three groups: ≥ 1500, 600 - 1500, and < 600 MET minutes per week (used as the reference group).

### 3.4. Definition of Metabolic Syndrome 

The Joint Interim Statement (JIS) definition of MetS (3) has been applied in the current study. The presence of three or more of the following criteria is considered MetS: 

(1) High WC: WC ≥ 95 cm among Iranians (25) (≥ 88 cm in men and ≥ 102 cm in women in the American population). 

(2) High TG: TG ≥ 150 mg/dL or use of lipid-lowering medications. 

(3) Low HDL-C: HDL-C < 40 mg/dL for males and < 50 mg/dL for females, or use of medications for reduced HDL-C. 

(4) High blood pressure: SBP/DBP ≥ 130/85 mmHg or use of antihypertensive medications. 

(5) High FPG: FPG ≥ 100 mg/dL or use of anti-diabetic medications.

### 3.5. Statistical Analysis 

The mean ± standard deviation (SD) and number (%) for categorical variables were used to present the baseline characteristics of the study population. For covariates with a skewed distribution, the median interquartile range (IQR) was reported. A comparison of baseline characteristics between normal and abnormal ALT, as defined by two different criteria, was conducted using the Student’s *t*-test for continuous variables, the chi-square test for categorical variables, and the Mann-Whitney test for skewed variables.

The association between ALT level (per 5 U/L) and MetS and its components was assessed by calculating multivariable-adjusted odds ratios (ORs) with a 95% confidence interval (CI) using binary logistic regression analysis. Confounding variables were adjusted for in four models as follows: Model 1 was a univariable model that included only ALT. Model 2 was adjusted for age, and Model 3 included further adjustments for education level, physical activity, and smoking status. Additionally, the area under the curve (AUC) with a 95% CI was calculated to assess the discriminative ability of ALT for MetS .

To determine the cut-off value of ALT for predicting the composite outcome, Youden’s index (16) was calculated as [Y = sensitivity – (1 – specificity)], and the maximum value was considered the optimal ALT cut-off point. Diagnostic performance indicators, including sensitivity, specificity, and positive and negative predictive values, were calculated for the cut-off points derived for MetS . Furthermore, we reported the diagnostic performance of thresholds recommended by the National Health and Nutrition Examination Survey in the United States (US-NHANES) (ALT ≥ 30 U/L for females and ≥ 40 U/L for males) (26, 27), abnormal ALT levels (ALT > 19 U/L for females and > 30 U/L for males) for liver-related mortality in the US population (US-LRM), as recommended by Ruhl CE and Everhart JE (28), and previous Iranian studies (north Iranian population; ALT > 18.8 U/L for females and > 21.4 U/L for males) (29). 

As part of the external validation, the diagnostic performance of various cut-off points was assessed using the ARIC dataset. We also excluded adults on anti-diabetic medications as a sensitivity analysis to assess the robustness of the results. 

All analyses were performed separately by gender. All analyses were conducted using STATA version 17 SE (StataCorp, TX, USA), and a two-tailed P-value < 0.05 was considered significant.

## 4. Results 

The baseline characteristics of TLGS participants according to MetS status for each gender are shown in Table 1. There were significant differences in baseline characteristics between those with and without MetS, except for physical activity among women. In both genders, compared to participants without MetS, those with MetS were older and had higher values for BMI, WC, SBP, DBP, FPG, TG, and ALT, and lower levels of HDL-C. Additionally, they were more likely to be married and had a higher education level (6 - 12 years). Furthermore, male participants with MetS were more likely to smoke and had lower levels of physical activity compared to those without Mets.

**Table 1.** Baseline Characteristics of the Study Population According to the Metabolic Syndrome Groups Among Men and Women Separately: Tehran Lipids and Glucose Study ^a^

Variables	Women			Men		
	Without MetS (n = 1750)	With MetS (n = 982)	P-Value	Without MetS (n = 1195)	With MetS (n = 1041)	P-Value
**Age, y**	41.0 ± 11.4	52.5 ± 10.1	< 0.001	40.0 ± 12.7	46.8 ± 11.6	< 0.001
**BMI, kg/m** ^**2**^	26.2 ± 4.6	31.2 ± 4.8	< 0.001	26.0 ± 4.3	29.5 ± 4.3	< 0.001
**WC, cm**	89.2 ± 10.5	101.6 ± 10.1	< 0.001	92.2 ± 11.1	101.7 ± 9.8	< 0.001
**SBP, mmHg**	102.6 ± 11.2	116.2 ± 15.1	< 0.001	111.1 ± 11.8	120.3 ± 14.8	< 0.001
**DBP, mmHg**	70.7 ± 7.4	77.0 ± 8.7	< 0.001	75.6 ± 8.1	82.1 ± 9.7	< 0.001
**FPG, mg/dL**	91.5 ± 13.6	111.4 ± 37.4	< 0.001	94.5 ± 21.2	110.4 ± 37.0	< 0.001
**TG, mg/dL, median (IQR)**	96 (72 - 125)	165 (123 - 211)	< 0.001	107 (81 - 139)	189 (149 - 255)	< 0.001
**HDL-C, mg/dL**	53.1 ± 10.2	46.1 ± 10.4	< 0.001	46.4 ± 9.0	37.9 ± 8.9	< 0.001
**ALT, U/L**	16.7 ± 9.4	22.1 ± 13.3	< 0.001	26.2 ± 19.3	31.8 ± 19.1	< 0.001
**Marital status**			< 0.001			< 0.001
Single	376 (21.5)	55 (5.6)		426 (35.6)	147 (14.1)	
Married	1250 (71.4)	798 (81.3)		743 (62.2)	872 (83.8)	
Widow**/**divorced	124 (7.1)	129 (13.1)		26 (2.2)	22 (2.1)	
**Education levels, y**			< 0.001			< 0.001
< 6	61 (3.5)	151 (15.4)		34 (2.8)	27 (2.6)	
6 - 12	741 (42.3)	559 (56.9)		551 (46.1)	528 (50.7)	
≥ 12	948 (54.2)	272 (27.7)		610 (51.1)	486 (46.7)	
**Smoking status**			< 0.001			0.007
None	1363 (77.9)	840 (85.5)		476 (39.8)	423 (40.6)	
Current	51 (2.9)	29 (2.9)		135 (11.3)	160 (15.4)	
Past	336 (19.2)	113 (11.5)		584 (48.9)	458 (44.0)	
**Anti-diabetic medications**	32 (1.8)	240 (24.4)	< 0.001	18 (1.5)	132 (12.7)	< 0.001
**High blood pressure medications**	37 (2.1)	283 (28.8)	< 0.001	31 (2.6)	170 (16.3)	< 0.001
**Lipid-lowering medications **	26 (1.5)	378 (38.5)	< 0.001	11 (1.0)	196 (18.8)	< 0.001

Abbreviations: BMI, Body Mass Index; WC, waist circumference; SBP, systolic blood pressure; DBP, diastolic blood pressure; FPG, fasting plasma glucose; HDL-C, high density lipoprotein cholesterol; TG, triglycerides; ALT, alanine transaminase; MetS, metabolic syndrome.

^a^ Values are expressed as mean ± SD or No. (%) unless otherwise indicated.

Table 2 shows the sex-specific adjusted ORs and 95% CI for MetS and its components per 5 U/L increase in ALT in different populations. In the TLGS, each 5 U/L increase in ALT level was associated with a 15% and 26% higher chance of having MetS after adjusting for age, education level, physical activity, and smoking habits in males and females, respectively. The corresponding values for the ARIC data were 25% and 20%, respectively. Moreover, the same association was observed between MetS components and each 5 U/L increase in ALT level.

**Table 2.** Association Between Alanine Transaminase (per 5 U/L) with Prevalent Metabolic Syndrome and Its Components Among Iranian and American Populations ^a,^ ^b^

Variables	Women		Men	
	TLGS	ARIC	TLGS	ARIC
	OR (95 % CI)	OR (95 % CI)	OR (95 % CI)	OR (95 % CI)
**MetS**	E/N = 982/2732	E/N = 2429/6306	E/N = 1041/2236	E/N = 2998/4841
Model 1	1.28 (1.23 - 1.34)	1.19 (1.15 - 1.23)	1.09 (1.07 - 1.12)	1.23 (1.18 - 1.28)
Model 2	1.25 (1.19 - 1.31)	1.20 (1.16 - 1.24)	1.15 (1.11 - 1.18)	1.26 (1.21 - 1.31)
Model 3	1.26 (1.20 - 1.32)	1.20 (1.16 - 1.24)	1.15 (1.11 - 1.18)	1.25 (1.21 - 1.31)
**Elevated WC**	E/N = 1702/2732	E/N = 2107/6306	E/N = 1697/2236	E/N = 4337/4841
Model 1	1.30 (1.24 - 1.37)	1.16 (1.13 - 1.20)	1.15 (1.11 - 1.20)	1.43 (1.33 - 1.54)
Model 2	1.23 (1.17 - 1.30)	1.17 (1.13 - 1.21)	1.19 (1.14 - 1.24)	1.45 (1.35 - 1.57)
Model 3	1.24 (1.18 - 1.31)	1.16 (1.12 - 1.20)	1.19 (1.14 - 1.24)	1.41 (1.31 - 1.52)
**Elevated pressure**	E/N = 524/2732	E/N = 3012/6306	E/N = 685/2236	E/N = 2250/4841
Model 1	1.10 (1.06 - 1.14)	1.08 (1.05 - 1.11)	1.03 (1.00 - 1.05)	1.06 (1.03 - 1.09)
Model 2	1.07 (1.02 - 1.12)	1.08 (1.05 - 1.12)	1.06 (1.03 - 1.09)	1.09 (1.06 - 1.12)
Model 3	1.07 (1.03 - 1.12)	1.08 (1.04 - 1.09)	1.06 (1.04 - 1.09)	1.09 (1.06 - 1.12)
**Elevated FPG**	E/N = 789/2732	E/N = 3510/6306	E/N = 761/2236	E/N = 3382/4841
Model 1	1.21 (1.16 - 1.26)	1.16 (1.12 - 1.20)	1.03 (1.01 - 1.05)	1.12 (1.08 - 1.16)
Model 2	1.18 (1.13 - 1.23)	1.16 (1.12 - 1.20)	1.08 (1.05 - 1.11)	1.13 (1.09 - 1.18)
Model 3	1.18 (1.13 - 1.23)	1.16 (1.12 - 1.20)	1.08 (1.05 - 1.11)	1.13 (1.09 - 1.17)
**Elevated TG**	E/N = 1019/2732	E/N = 1905/6306	E/N = 1099/2236	E/N = 1727/4841
Model 1	1.24 (1.19 - 1.29)	1.11 (1.07 - 1.14)	1.11 (1.08 - 1.14)	1.18 (1.14 - 1.21)
Model 2	1.20 (1.15 - 1.25)	1.11 (1.08 - 1.14)	1.14 (1.11 - 1.17)	1.18 (1.14 - 1.22)
Model 3	1.20 (1.15 - 1.26)	1.11 (1.08 - 1.15)	1.14 (1.11 - 1.17)	1.18 (1.14 - 1.22)
**Reduced HDL-C**	E/N = 1508/2732	E/N = 2641/6306	E/N = 1021/2236	E/N = 2331/4841
Model 1	1.11 (1.07 - 1.15)	1.11 (1.08 - 1.15)	1.05 (1.02 - 1.08)	1.10 (1.06 - 1.13)
Model 2	1.09 (1.05 - 1.14)	1.12 (1.08 - 1.15)	1.07 (1.04 - 1.09)	1.10 (1.07 - 1.13)
Model 3	1.09 (1.05 - 1.14)	1.13 (1.09 - 1.16)	1.06 (1.04 - 1.09)	1.11 (1.07 - 1.14)

Abbreviations: E, event; N, number of population; OR, odds ratio; CI, confidence interval; ALT, alanine transaminase; BMI, Body Mass Index; TLGS, Tehran lipid and glucose study; ARIC, atherosclerosis risk in communities; MetS, metabolic syndrome; WC, waist circumference; DBP, diastolic blood pressure; HDL-C, high density lipoprotein cholesterol; FPG, fasting plasma glucose.

^a^ Elevated WC: ≥ 95 cm for Iranians (≥ 88 cm for men and ≥ 102 cm for women in the American population); elevated TG or drug treatment for elevated TG: ≥ 150 mg/dL; reduced HDL-C or drug treatment for reduced HDL-C: < 40 mg/dL for males and < 50 mg/dL for females; elevated blood pressure or antihypertensive drug treatment in a patient with a history of hypertension: SBP/DBP ≥ 130/85 mmHg; elevated FPG or drug treatment for elevated glucose: ≥ 100 mg/dL.

^b^ Model 1: Only ALT; model 2: Model 1 + age; model 3: Model 2 + education levels + physical activity + smoking status.

As shown in Table 3, the optimal cut-off point for ALT, supported by Youden's Index for screening MetS , was 21.3 U/L in males, which showed a sensitivity of 72.1% and specificity of 47.1%. The corresponding values for the US-LRM suggested threshold in the US population (30 U/L) were 32.1% and 78.8%, respectively. Among females, the new cut-off point was 17.6 U/L, showing a sensitivity of 57.9% and specificity of 66.5%. The corresponding values for the LRM threshold in the US population (19 U/L) were 50.8% and 71.5%, respectively.

**Table 3.** Diagnostic Tests of Different Alanine Transaminase Suggested Cut-Off Points for Prevalent Metabolic Syndrome Among Men and Women Separately: Tehran Lipids and Glucose Study (2018 - 2022)

Variables	Cut-Point (U/L)	High-Risk Population	Event (%)	Sensitivity (%)	Specificity (%)	PPV (%)	NPV (%)	AUC (95% CI)	OR ^a^ (95% CI)
**Females (n = 2732)**									
Currents study	17.6	1156	49.2	57.9	66.5	49.2	73.8	0.62 (0.60 -0.64)	2.21 (1.82 - 2.68)
North Iranian population (29)	18.8	1013	50.2	51.8	71.2	50.2	72.5	0.61 (0.59 - 0.63)	2.16 (1.06 - 1.09)
US-LRM (28)	19.0	997	50.1	50.8	71.5	50.1	72.2	0.61 (0.59 - 0.63)	2.15 (1.77 - 2.61)
US-NHANCE (26)	31.0	231	60.6	14.3	94.8	60.6	65.8	0.54 (0.53 - 0.56)	2.30 (1.66 - 3.18)
**Males (n = 2236)**									
Currents study	21.3	1382	54.3	72.1	47.1	54.3	65.9	0.60 (0.58 - 0.61)	2.36 (1.91 - 2.91)
North Iranian population (29)	21.4	1368	54.3	71.4	47.7	54.3	65.7	0.59 (0.58 - 0.61)	2.33 (1.89 - 2.87)
US-LRM (28)	30.0	822	54.5	43.0	68.7	54.1	56.7	0.56 (0.54 - 0.58)	1.66 (1.36 - 2.04)
US-NHANCE (26)	40.0	386	59.8	22.2	87.0	59.8	56.2	0.55 (0.53 - 0.56)	1.85 (1.43 - 2.38)

Abbreviations: PPV, positive predictive value; NPV, negative predictive value; AUC, area under the curve; OR, odds ratio; LRM, liver related mortality; US-NHANCE, National Health and Nutrition Examination Survey in the United States (US-NHANCE).

^a^ The model included r ALT, age, education levels, smoking status, and BMI.

Moreover, compared to the US-LRM thresholds, the discrimination index of our suggested threshold increased in males (60% vs. 56%, respectively), while for females, it remained the same as in the pretest (≈ 62%). The suggested cut-off point was significantly better than those calculated using the US-NHANCE thresholds.

The diagnostic values of different cut points for screening MetS using ARIC data are listed in Table 4 Compared to the US-LRM threshold among females, our suggested threshold showed almost the same AUC values (58% vs. 57%, respectively), but it was higher than the US-NHANCE threshold (58% vs. 56%, respectively). Among males, the highest AUC was observed for our suggested cut-off points (56%), followed by the US-LRM thresholds (53%), and the US-NHANCE threshold (51%).

**Table 4.** External Validation of a Diagnostic Test of Different Alanine Transaminase Suggested Cut-Off Points for Prevalent Metabolic Syndrome Among Men and Women Separately, using ARIC data (1990 - 1992)

Variables	Cut-Point (U/L)	High-Risk Population	Event (%)	Sensitivity (%)	Specificity (%)	PPV (%)	NPV (%)	AUC (95% CI)	OR ^a^ (95% CI)
**Females (n = 6306)**									
Currents study	17.6	1503	54.0	33.4	82.1	54.0	66.3	0.58 (0.56 - 0.59)	1.87 (1.63 - 2.15)
US-LRM (28)	19.0	1272	55.3	29.0	85.3	55.3	63.4	0.57 (0.56 - 0.58)	1.89 (1.63 - 2.19)
US-NHANCE (26)	31.0	234	58.6	5.6	97.5	58.5	62.2	0.52 (0.51 - 0.52)	1.73 (1.27 - 2.35)
**Males (n = 4841)**									
Currents study	21.3	1265	73.6	31.0	81.9	73.6	42.2	0.56 (0.55 - 0.58)	1.65 (1.41 - 1.92)
US-LRM (28)	30.0	512	76.7	13.1	93.5	76.8	39.8	0.53 (0.52 - 0.54)	1.78 (1.41 - 2.25)
US-NHANCE (26)	40.0	143	79.0	3.8	98.4	79.0	39.8	0.51 (0.51 - 0.52)	1.97 (1.27 - 3.06)

Abbreviations: PPV, positive predictive value; NPV, negative predictive value; AUC, area under the curve; OR, odds ratio; CI, confidence interval; ALT, alanine transaminase; BMI, Body Mass Index; LRM, liver related mortality; US-NAHANCE, National Health and Nutrition Examination Survey in the United States (US-NHANCE).

^a^ Adjusted for ALT, age, education levels, smoking status, and BMI.

As a sensitivity analysis, we excluded 442 adults on anti-diabetic medications from the TLGS data and 664 individuals from the ARIC data. The results were largely consistent. When considering the optimal cut-off point analysis, the recommended value for females decreased to 14.5 U/L, with a sensitivity of 73.8% and specificity of 50.5%, yet the discrimination power remained unchanged. For males, the results were consistent with the main analysis (Appendices 1 and 2 in Supplementary File).

## 5. Discussion 

The present study aimed to investigate the association between ALT levels and the prevalence of MetS among the Iranian population and to externally validate the findings in the American population. The dose-response analysis revealed a notable increase in the prevalence of MetS with each 5 U/L increase in ALT levels, resulting in a 20% increase among females and a 15% increase among males. Furthermore, the gender-specific ALT cut-off points for screening MetS , supported by Youden's Index, were found to be 21 U/L for males and 18 U/L for females. Notably, these suggested cut-off points exhibited a similar discrimination index AUC to the American cut-off points for females, but superior discrimination for males in both the Iranian and American populations. This improvement was achieved by increasing sensitivity while reducing specificity.

Our findings were consistent with a comprehensive meta-analysis of seven prospective studies involving 31,545 individuals, which reported that for every 5 U/L rise in ALT levels, the relative risk (RR) of incident MetS increased by 13% in males and 38% in females (gender difference P-value = 0.007) (16). This gender discrepancy could be attributed to the higher prevalence of MetS among females compared to males (30, 31).

Previous studies have consistently demonstrated that ALT levels are higher in males compared to females (20, 29, 32), a finding also observed in our current study. This gender disparity suggests the need for gender-specific cut-off points when interpreting ALT values and their potential association with the prevalence of MetS. In our study, we identified optimal suggested cut-off values for ALT: 18 U/L for females and 21 U/L for males. These cut-off values exhibited higher sensitivity for males and higher specificity for females. Our findings align closely with the cut-off values derived from a prior population study conducted in Iran (29). However, it is important to note that the recommended cut-off values for elevated ALT levels in the US-NHANES (for both genders) (27) and those suggested by Ruhl and Everhart for LRM (28) (for males) had lower discriminatory power compared to our suggested thresholds. Using the US-LRM threshold level suggested by Ruhl and Everhart (28), a specificity of about 70% was achieved for both men and women. Similarly, the corresponding specificity using the US-NHANES threshold was 95% for females and 87% for males.

Furthermore, to evaluate the applicability of our suggested cut-off points in the Iranian population, we conducted an external validation using data from the ARIC Study, which represents a middle-aged US population. While the discrimination value of the defined cut-off point in the US population is similar to the results we observed in females, it significantly decreases in males, leading to a substantially lower AUC value compared to the AUC level established by our defined cut-off point.

Several limitations of the current study need to be considered. First, since it was a cross-sectional study, we were unable to determine the cut-off point for incident MetS. Second, we did not measure markers of the Hepatitis B virus, although its prevalence is low, at about 2.2% among the Tehranian population (33). Third, this study was conducted among the Tehranian urban population, and the results may not be generalizable to rural individuals. However, despite this limitation, we conducted an external validation to assess the applicability of the derived thresholds among the American population, thereby enhancing the robustness and potential broader relevance of our results. Fourth, the discriminatory power of the suggested cut-off points for screening MetS is lower than the accepted value, and further research, particularly regarding the prognostic power for incident MetS, could improve predictive accuracy. Nevertheless, even a modest AUC can provide clinical diagnostic value by identifying individuals at risk for MetS and enabling targeted interventions.

### 5.1. Conclusions

In conclusion, ALT levels over 21 U/L for males and 18 U/L for females may be applicable for screening MetS in the Tehranian population. However, further research is needed to validate these findings in other populations and to explore the underlying mechanisms linking ALT levels to the development and progression of MetS.

ijem-23-1-151542-s001.pdf

## Data Availability

The dataset presented in the study is available upon request from the corresponding author during submission or after publication. The data are not publicly available due to restrictions from the Research Institute.
