# Supplementary material for: The Optimal Cut-Points of Alanine Aminotransferase for Screening Metabolic Syndrome in Iranian Adults
Source: Int J Endocrinol Metab. 2025 Jan 25;23(1):e151542. doi: 10.5812/ijem-151542 (PMC12118368; doi:10.5812/ijem-151542)
Supplement: ijem-23-1-151542-s001.pdf [file ijem-23-1-151542-s001.pdf]

Appendix 1: Association between alanine transaminase (per 5-U/L) with prevalent metabolic syndrome and elevated FPG after excluding those on anti-diabetic medications among Iranian and American populations				
	**women**		**Men**	
	**TLGS**	**ARIC**	**TLGS**	**ARIC**
	**OR (95 % CI)**	**OR (95 % CI)**	**OR (95 % CI)**	**OR (95 % CI)**
**METS**	**E/N=742/2460**	**E/N=2099/5914**	**E/N=909/2086**	**E/N=2752/4569**
**Model 1**	1.25(1.19-1.31)	1.19(1.15-1.23)	1.11(1.08-1.14)	1.23(1.18-1.28)
**Model 2**	1.22(1.16-1.28)	1.19(1.15-1.24)	1.15(1.11-1.18)	1.25(1.20-1.30)
**Model 3**	1.22(1.16-1.28)	1.19(1.15-1.24)	1.15(1.11-1.18)	1.25(1.20-1.30)
**Elevated FPG**	**E/N=789/2732**	**E/N=3118/5914**	**E/N=761/2236**	**E/N=3110/4569**
**Model 1**	1.17(1.12-1.22)	1.13(1.09-1.17)	1.04(1.02-1.07)	1.12(1.08-1.16)
**Model 2**	1.15(1.10-1.20)	1.14(1.10-1.18)	1.08(1.05-1.11)	1.13(1.09-1.18)
**Model 3**	1.15(1.10-1.20)	1.14(1.09-1.18)	1.08(1.05-1.11)	1.12(1.09-1.17)

E: event; N=number of population; OR: odds ratio; CI: confidence interval; ALT: alanine transaminase; BMI: body mass index;
Elevated FPG: elevated glucose: ≥100 mg/dl
Model 1: included only ALT
Model 2: model 1+age
Model 3: model 2+ education levels + physical activity+ smoking status

Appendix 2: Diagnostic test of a different alanine transaminase suggested cut-off points for prevalent metabolic syndrome after exc[illegible] diabetic medications among men and women separately: Tehran lipids and glucose study (2018-2022)

	Cut-point, U/L	High-risk population	Event, %	Sensitivity, %	Specificity, %	PPV, %	NPV, %	AUC (95[illegible]
**Females (n=2732)**								
- **Currents study**	14.5	1382	54.3	73.8	50.5	45.6	77.5	0.62(0.60[illegible]
**Males (n=2236)**								
- **Currents study**	21.3	1382	54.3	72.1	47.1	54.3	65.9	0.60(0.58[illegible]

PPV: positive predictive value; NPV: negative predictive value; AUC: Area under the curve; OR: odds ratio.
*Model included ALT, age, education levels, smoking status, and BMI
